# Diarrhea, negative t-waves, fever and skin rash, rare manifestation of carbamazepine hypersensitivity: a case report

**DOI:** 10.1186/1757-1626-1-312

**Published:** 2008-11-14

**Authors:** Felix Aigner, Wolfgang Aigner, Friedrich Hoppichler, Gerhard Luef, Hugo Bonatti

**Affiliations:** 1 Department of Visceral, Transplant and Thoracic Surgery, Innsbruck Medical University, Anichstrasse 35, A-6010 Innsbruck, Austria; 2 General Practitioner, Walserfeldstrasse 181, A-5071 Wals, Austria; 3 Hospital of the Barmherzige Brueder Salzburg, Department of Medicine, Kajetanerplatz 1, A-5010 Salzburg, Austria; 4 Department of Neurology, Innsbruck Medical University, Anichstrasse 35, A-6010 Innsbruck, Austria; 5 Department of Surgery, University of Virginia health services, Charlottesville, VA, USA

## Abstract

**Introduction:**

Antiepileptic drug induced hypersensitivity syndrome is a rare side effect of some of the first line anticonvulsive drugs such as carbamazepine and other aromatic agents. We are the first to mention a rare case of gastrointestinal, skin and cardiac findings related to carbamazepine administration, which is very uncommon and needs to be reported.

**Case presentation:**

We report on a 62-year-old Caucasian woman with carbamazepine associated hypersensitivity syndrome, who developed diarrhea, fever, skin lesions, pericardial effusion and pathology on electrocardiogram with terminal negative T waves in I, II, aVL, V_5 _and V_6_,. After withdrawal of carbamazepine and administration of methylprednisolone, all initial symptoms improved, white blood cell count normalized, pericardial effusion resolved and pathologic electrocardiogram findings resolved.

**Conclusion:**

Anticonvulsive drug hypersensitivity syndromes can present with a wide spectrum of unspecific symptoms, which the prescribing clinician should be aware of.

## Introduction

Antiepileptic drug induced hypersensitivity syndrome (AEDHS) is a rare side effect of some of the first line anticonvulsive drugs such as carbamazepine (CBZ) and other aromatic agents. Typically AEDHS presents with skin lesions, fever and lymphadenopathy; in more severe cases also internal organs may be involved [1-6].

CBZ is administered for the treatment of simple and complex epileptic seizures, trigeminal neuralgia and alcohol withdrawal syndrome. The drug has anticonvulsive and anticholinergic properties by reducing excessive nerve signals in the brain and restoring the normal balance of nerve activity. Side effects include diarrhea and colitis, aplastic anemia and agranulocytosis as well as other rare conditions such as cardiotoxicity [7,8].

Due to this broad spectrum of side effects, differentiation from AEDHS may be difficult, but CBZ has been linked to AEDHS affecting the liver, lungs and kidneys [1-6,9]. We report on a patient with CBZ associated AEDHS who developed diarrhea, fever, skin lesions and pathology on electrocardiogram.

## Case presentation

A 62-year-old Caucasian woman, 5.4 ft and 165 lbs, with no history of drug use was admitted to a neurological emergency unit after a first epileptic seizure event reporting of monthly recurrent ill temper. She reported past cholecystectomy 2001, positive family history for prostate cancer (father) and Alzheimer's disease (mother). The patient had two uncomplicated vaginal deliveries, no smoking history and alcohol consumption occasionally. No medication separate to this case. Neurological workup included EEG, CT of the brain, MRT but no intracranial pathology could be demonstrated. Blood and CSF chemistry were unremarkable and serum as well as CSF tested negative for bacterial, viral, fungal or parasitic pathogens, Thus it was suspected that the patient suffered from epilepsia and abdominal pain was thought to represent an epigastric aura. Treatment with CBZ at a dose of 200 mg twice daily was started.

Ten days after introduction of CBZ the patient developed a reddish maculo-papulous rash with pruritus affecting her entire body except the face and legs; simultaneously, she developed fever and watery diarrhea. CBZ was decreased to 200 mg once daily, antihistaminic and antipyretic drugs were started, which resulted in an improvement of symptoms (temperature normalized, skin lesions disappeared and diarrhea improved). The plasma CBZ level was 3.0 mg/L (therapeutic range 4 to 12 mg/L).

**Figure 1:**
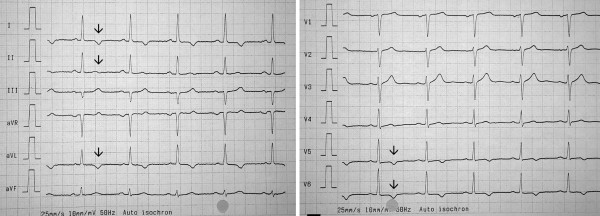
12-lead electrocardiogram (ECG) three weeks after carbamazepine (CBZ) withdrawal. Arrows indicate negative T waves in I, II, aVL, V_5 _and V_6_.

Twenty days later the patient's condition dramatically worsened. The exanthema reappeared and became generalized, she developed again watery diarrhea and her temperature rose to 39 degrees centigrade. She was admitted to hospital and CBZ was replaced with valproic acid (VPA). Laboratory findings showed a normal white blood cell count with relative eosinophilia (8% of total leukocytes) and elevated transaminases (AST of 45 U/L, ALT of 53 U/L, γGT of 65 U/L). C-reactive protein (CRP) level was 2.2 mg/dL. Serum creatinine was elevated to 1.3 mg/dl and serum potassium level was increased at 5.5 mmol/L. Chest x-ray and abdomen ultrasound were unremarkable, but the electrocardiogram (ECG) showed terminal negative T waves in I, II, aVL, V_5 _and V_6 _(Fig. 1) with normalizing tendency after strain. An ischemic cardiac event was excluded by normal myocardial scintigraphy and negative angina history and normal troponine T and creatinine kinase (including myocardial specific CK). Transesophageal echocardiography showed no signs of myocardial hypertrophy, a good ventricular function but a small pericardial effusion. Stool cultures were negative for enteric pathogens and no occult blood could be detected.

**Figure 2:**
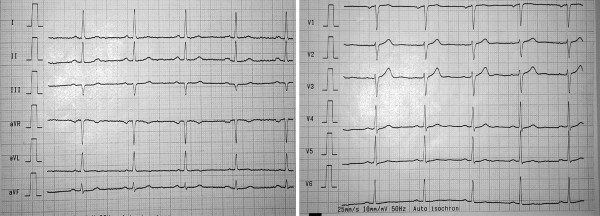
12-lead electrocardiogram (ECG)five weeks after carbamazepine (CBZ) withdrawal. Arrows indicate negative T waves in I, II, aVL, V_5 _and V_6_.

Following withdrawal of CBZ and application of corticosteroids (i.v. methylprednisolone at a dose of 16 mg daily for one weeks) and antihistamines, the patients condition rapidly improved, diarrhea stopped, she defeveresced and the ECG normalized (Fig. 2).

## Discussion

Our patient presented with classic symptoms of AEDHS including rash, fever, diarrhea and elevated liver enzymes ten days after initiation of CBZ therapy. Following temporary improvement after lowering the dose symptoms worsened again and the patient required hospitalisation. She had no history of drug related side effects, gastrointestinal, dermatological or cardiac disorders and no apparent acute infection making AEDHS a plausible diagnosis. The changes in the ECG and the pericardial effusion have not been associated with AEDHS, but we believe that either CBZ or metabolites were directly causing these complications. Alternatively, it could be the secondary effect of hepatic and renal involvement of AEDHS. Terminal negative T waves and pericardial effusion are unspecific symptoms and may be triggered by various causes. There was no evidence for ischemia in our patient and after application of steroids and withdrawal of CBZ these pathologies normalized. Therefore, we believe that the cardiac pathology was a rare manifestation of AEDHS. Of note, other ECG changes have been previously observed after exposure to CBZ and included sinus bradycardia, atrioventricular blocks and myocarditis [10]. In contrast to a previous study from Serbia, where arrhythmia was observed in the course of acute CBZ poisoning [11], CBZ levels were within normal range throughout the treatment course in our case. Therefore, we assume a rare manifestation of AEDHS rather than cardiotoxicity in our patient. AEDHS can present with a wide spectrum of symptoms such as skin lesions, fever, lymphadenopathy in combination with pathologic laboratory findings such as leucocytosis/leucopenia and elevated liver enzymes. It has been described after exposure to aromatic drugs such as CBZ, phenytoin and Phenobarbital [1-6]. Onset of AEDHS may develop one week to three months after CBZ administration [3], the term "drug rash with eosinophilia and systemic symptoms" (DRESS) has been previously used [3,12,13]. The exact mechanism of AEDHS remains to be determined but is thought to be associated with enzymatic deficiency or abnormality in the metabolism of anticonvulsants, reactivation of herpes-type viruses, and ethnic predisposition with certain human leukocyte antigen subtypes [14]. Internal organ involvement affecting liver, lungs, lymphatic system and kidneys may lead to misinterpretation of the symptoms [1-6]. CBZ is well known to cause neutropenia and pancytopenia. Up to 1–2 cases per year per 100.000 subjects may develop agranulocytosis as the worst complication [15]. Individuals with "slow liver metabolism" may be at increased risk to develop "drug induced hypersensitivity syndrome" due to the production of toxic metabolites in the liver even after CBZ has been completely cleared; CBZ has also been linked to acute liver failure. Enterocolitis may be caused by various antiepileptic drugs [7,8]. Diagnosis of enteric manifestation of AEDHS is based on a close temporal relationship between drug exposure and development of diarrhea as well as improvement after withdrawal of the causing drug like in our case. The role of glucocorticoids in the treatment of AEDHS has been controversially discussed [3]. Glucocorticoids decrease the levels of IL-5, which is one of the major growth factors for eosinophilic granulocytes (especially in eosinophilic conditions). In our patient, methylprednisolone was used, which resulted in an improvement in all initial symptoms and normalization of the previously elevated white blood cell count. Most importantly the pericardial effusion resolved and pathologic ECG findings (terminal negative T waves in I, II, aVL, V_5 _and V_6_,) normalized.

## Consent

Written informed consent was obtained from the patient for publication of this case report and accompanying images. A copy of the written consent is available for review by the Editor-in-Chief of this journal.

## Abbreviations

AEDHS: Antiepileptic drug induced hypersensitivity syndrome; CBZ: carbamazepine; EEG: electro-encephalogram; CT: computed tomography; MRT: magnetic resonance tomography; CSF: cerebrospinal fluid; VPA: valproic acid; CRP: C-reactive protein; ECG: electrocardiogram; CK: creatinine kinase; AST: aspartat-aminotransferase; ALT: alanin-aminotransferase; γGT: gamma-glutamyl-transferase; DRESS: drug rash with eosinophilia and systemic symptoms; IL-5; interleukin-5.

## Competing interests

The authors declare that they have no competing interests.

## Authors' contributions

FA and HB analyzed and interpreted the patient data and were major contributors in writing the manuscript. WA is the general practitioner of the patient and cares for the patient in the long-term follow-up. FH is head of the Department of Internal Medicine where the patient was basically treated. GL interpreted the patient data regarding the neurological disorder. All authors read and approved the final manuscript.

## References

[B1] Shear NH, Spielberg SP (1988). Anticonvulsant hypersensitivity syndrome: in vitro assessment of risk. J Clin Invest.

[B2] Handfield-Jones SE, Jenkins RE, Whittaker SJ, Besse CP, McGibbon DH (1993). The anticonvulsant hypersensitivity syndrome. Br J Dermatol.

[B3] Valencak J, Ortiz-Urda S, Heere-Ress E, Kunstfeld R, Base W (2004). Carbamazepine-induced DRESS syndrome with recurrent fever and exanthema. Int J Dermatol.

[B4] Schlienger RG, Shear NH (1998). Antiepileptic drug hypersensitivity syndrome. Epilepsia.

[B5] Ray-Chaudhuri K, Pye IF, Boggild M (1989). Hypersensitivity to carbamazepine presenting with a leukemoid reaction, eosinophilia, erythroderma and renal failure. Neurology.

[B6] De Vriese AS, Philippe J, Van Renterghem DM, De Cuyper CA, Hindryckx PH, Matthys EG, Louagie A (1995). Carbamazepine hypersensitivity syndrome: report of 4 cases and review of the literature. Medicine (Baltimore).

[B7] Eland IA, Dofferhoff AS, Vink R, Zondervan PE, Stricker BH (1999). Colitis may be part of the antiepileptic drug hypersensitivity syndrome. Epilepsia.

[B8] Bosman T, Vonck K, Claeys P, Van Vlierberghe H, De Clercq M, De Reuck J, Boon P (2004). Enterocolitis: an adverse event in refractory epilepsy patients treated with levetiracetam?. Seizure.

[B9] Chong SA, Mythily, Mahendran R (2001). Cardiac effects of psychotropic drugs. Ann Acad Med Singapore.

[B10] Salzman MB, Valderrama E, Sood SK (1997). Carbamazepine and fatal eosinophilic myocarditis. N Engl J Med.

[B11] Todoroviæ V, Randeloviæ S, Joksoviæ D, Joviæ-Stosiæ J, Vuciniæ S, Glisoviæ L (1993). Carbamazepine cardiotoxicity in acute poisoning. Vojnosanit Pregl.

[B12] Bocquet H, Bagot M, Roujeau JC (1996). Drug-induced pseudolymphoma and drug hypersensitivity syndrome (Drug Rash with Eosinophilia and Systemic Symptoms: DRESS). Semin Cutan Med Surg.

[B13] Tas S, Simonart T (2003). Management of drug rash with eosinophilia and systemic symptoms (DRESS syndrome): an update. Dermatology.

[B14] Bohan KH, Mansuri TF, Wilson NM (2007). Anticonvulsant hypersensitivity syndrome: implications for pharmaceutical care. Pharmacotherapy.

[B15] Oyesanmi O, Kunkel EJ, Monti D, Field H (1999). Hematologic side effects of psychotropics. Psychosomatics.

